# Matrix factorization for the reconstruction of cervical cancer screening histories and prediction of future screening results

**DOI:** 10.1186/s12859-022-04949-8

**Published:** 2022-11-16

**Authors:** Geir Severin R. E. Langberg, Mikal Stapnes, Jan F. Nygård, Mari Nygård, Markus Grasmair, Valeriya Naumova

**Affiliations:** 1 Department of Research, Cancer Registry of Norway, Ullernchausseen 64, 0379 Oslo, Norway; 2 Department of Registry Informatics, Cancer Registry of Norway, Ullernchausseen 64, 0379 Oslo, Norway; 3 Department of Mathematical Sciences, Norwegian University of Science and Technology, Høgskoleringen 1, 7491 Trondheim, Norway; 4 Department of Machine Intelligence, SimulaMet, Pilestredet 52, 0167 Oslo, Norway

**Keywords:** Cervical cancer, Cancer screening, Population-level cancer prevention, Matrix completion, Matrix factorization

## Abstract

**Background:**

Mass screening programs for cervical cancer prevention in the Nordic countries have strongly reduced cancer incidence and mortality at the population level. An alternative to the current mass screening is a more personalised screening strategy adapting the recommendations to each individual. However, this necessitates reliable risk prediction models accounting for disease dynamics and individual data. Herein we propose a novel matrix factorisation framework to classify females by the time-varying risk of being diagnosed with cervical cancer. We cast the problem as a time-series prediction model where the data from females in the Norwegian screening population are represented as sparse vectors in time and then combined into a single matrix. Using novel temporal regularisation and discrepancy terms for the cervical cancer screening context, we reconstruct complete screening profiles from this scarce matrix and use these to predict the next exam results indicating the risk of cervical cancer. The algorithm is validated on both synthetic and registry screening data by measuring the *probability of agreement* (PoA) between Kaplan-Meier estimates.

**Results:**

In numerical experiments on synthetic data, we demonstrate that the novel regularisation and discrepancy term can improve the data reconstruction ability as well as prediction performance over varying data scarcity. Using a hold-out set of screening data, we compare several numerical models and find that the proposed framework attains the strongest PoA. We observe strong correlations between the empirical survival curves from our method and the hold-out data, and evaluate the ability of our framework to predict the females’ next results for up to five years ahead in time using only their current screening histories as input.

**Conclusions:**

We have proposed a matrix factorization model for predicting future screening results and evaluated its performance in a female cohort to demonstrate the potential for developing prediction models for more personalized cervical cancer screening.

## Background

The mass screening programs against cervical cancer established in the Nordic countries may have prevented up to 80 % of malignancies [1]. Persistent Human papillomavirus (HPV) infection is the primary causes of cervical cancer – as well as several other cancer types – initiating a process of cellular changes from low-grade to high-grade (pre-cancerous) lesions to invasive cancer [2]. Early detection of pre-cancerous lesions, e.g. with cytology, histology, or HPV tests, could prevent cancer development if it is treated [3] and motivates the need for screening.

A key factor in the success of the cancer screening programs is repeated screening at regular intervals. However, the risk of being infected with HPV and the risk of progressing to cancer vary significantly between females [4]. Thus, too frequent screening may lead to over-treatment of clinically insignificant pre-cancers, while too infrequent screening risks missing pre-cancers warranting treatment.

An alternative to the current mass-screening is a more personalized strategy adapting the screening frequency to the individual risk of disease initiation. For instance, vaccination of adolescent females has shown to improve protection against HPV infection [5], in which case the cancer screening programs may benefit from more flexible guidelines for the individual risk [6]. A step towards guidelines for more personalized recommendations is developing prediction models for the time-varying risk of cervical cancer using existing screening data from centrally organized population-level registries. In this paper, we present a novel matrix factorisation framework for time-dependent risk assessment of cervical cancer. We use population-based data from the *Norwegian Cervical Cancer Screening Program* (NCCSP) and evaluate our method by comparing Kaplan-Meier estimators from model predictions and a hold-out set.

The NCCSP database contains only the information needed by the *Cancer Registry of Norway* to administer the screening program. There are test results from 3 types of medical exams (cytology, histology, and HPV) but no further clinical information about the NCCSP participants. Following [7] we process these results into four *states*, reflecting the risk of cervical cancer and clinical consequences: A *normal* state indicates an accepted baseline risk; a *low-risk* state indicating an early stage of carcinogenesis (low-grade lesion) warranting more frequent screening to catch a potential progression to *high-risk*, requiring immediate treatment, and a *cancer* state, which can be seen as a failure of the screening program and a potentially lethal state for the woman.

In our approach we use NCCSP data collected between 1991–2015. During this time period, females aged 25–69 with a prior normal result were invited to a routine screening every 3rd year. According to those guidelines, triennial screening amounts to about 15 results in total and thus the state of the cervix is only observed at a few time points (*scarce* data). Moreover, since the recommendations are not strictly adhered to in practice the individual screening histories become *irregular* over time. Lastly, the majority of exam results are normal, making the data highly imbalanced. Specifically, in the NCCSP more than 90 % of test results are normal, 4–5 % low-risk and around 1 % are high-risk or cancer [8].

**Fig. 1:**
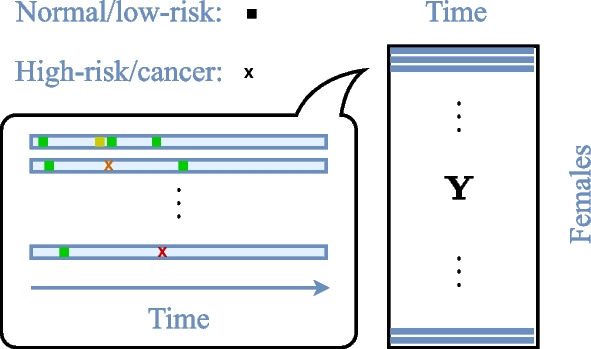
Matrix representation of cervical cancer screening histories. Individual cervical cancer screening histories as sparse time series fitted into a matrix ${\mathbf {Y}}$. A green/yellow square indicates normal/low-risk state, and an orange cross denotes a high-risk/cancer state. The matrix columns corresponds to female age intervals of 3 months

In Fig. 1, we illustrate screening histories represented by sparse time series vectors fitted into a matrix. Our goal is to estimate complete state profiles by filling the missing entries of these vectors and then use the completed state profiles in predicting the future state. Assuming correlation between subgroups of screening histories, we estimate the complete profiles using low-rank matrix factorisation (MF) and matrix completion (MC) techniques.

Existing methods applying MF to temporal data use similarity networks encoding temporal dependencies to facilitate constraints on the solution [9]. However, in our case the explicit temporal structure is not easily inferred from the data. Some recent work [10] extends the geometric deep learning (GDL) framework [11] to the matrix completion problem. Similarly to the temporal MF approaches, geometric deep learning methods also encode the structure of the data matrix using similarity graphs. The *PACIFIER* framework is a MF approach [12] specifically targeting the healthcare domain and the analysis of Electronic Medical Records, which can also be very sparse and noisy similar to the screening data. The PACIFIER performs MC by imposing sparsity and smoothness constraints on the temporal evolution of the latent factors.

In this paper, we adapt the PACIFIER framework to the cervical cancer screening setting and reconstruct complete state profiles from the scarce histories. We present a regulariser for the temporal dependencies between the results in histories and propose a discrepancy term for utilizing correlations between different histories. We evaluate our method on both synthetic data and registry data by measuring the *probability of agreement* [13] between Kaplan-Meier estimates from model predictions and a hold-out set.

## Results

In our experiments we consider five matrix factorization methods. The first method, referred to only as *matrix factorization* (MF), is our implementation of the PACIFIER. The second method, *convolutional* MF (CMF), extends the PACIFIER with more flexibility to model the variability observed in the cancer screening data. Furthermore, we introduce time shifts into the CMF to better exploit correlations between screening histories and name this *shifted* CMF (SCMF). We also consider versions of the CMF and SCMF where the errors in the discrepancy term are weighted to emphasize particular exam results. These models are referred to in our experiments as *weighted* CMF (WCMF) and *weighted* SCMF (WSCMF).

Moreover, we compare the matrix factorization models to the *GDL approach for matrix completion* (GDL) as in [10]. We studied different ways of constructing similarity graphs capturing the structure on the rows and columns of our matrix representation of screening histories, ${\mathbf {Y}}$, as input to GDL. Our strongest results over various distance metrics, including Euclidean and Wasserstein distance, came with a 10-NN sequential column graph for temporal smoothness and a 10-NN row graph based on the *cosine distance* to connect similar screening histories. Both graphs are weighted by $\exp (- d(i, j))$ with *d*(*i*, *j*) being the distance between two connected nodes *i* and *j*.

### Synthetic data experiments

**Fig. 2:**
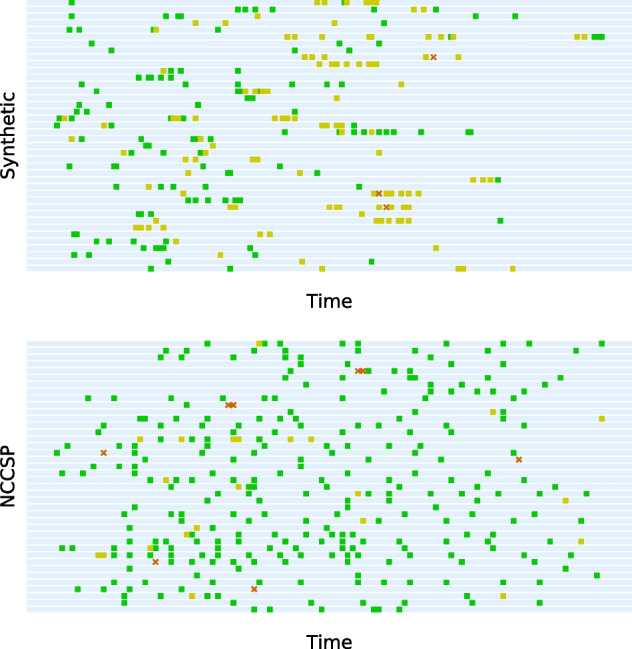
Comparing synthetic data to screening data. Randomly selected histories from synthetic data and data from the Norwegian Cervical Cancer Screening Programme (NCCSP). Green/yellow squares correspond to normal/low-risk results, and an orange cross signify either high-risk or cancer results

We generated synthetic data resembling the scarcity, irregularity and imbalance of the registry screening data. Latent state profiles were synthesized from linear combinations of five basic profiles of the form $V_{t, k} = \exp ( -10^{-3}(t - \mu _k)^2 )$ and female-specific coefficients $U_{n, k} \sim Exp(1)$. We mapped each of the entries in the latent state matrix ${\mathbf {M}} \in {\mathbb {R}}^{N \times T}$ to an integer 1–4 with model (2) at $\theta = 2.5$. Entries were randomly removed from the resulting integer matrix using empirical probabilities of observing an entry conditioned on the previous state. Figure 2 compares the synthetic data and the cancer screening registry data.

To measure the reconstruction error between the model estimate $\widehat{{\mathbf {M}}}$ and the ground truth ${\mathbf {M}}$ over the unobserved entries, we use

1 $$\begin{aligned} {\mathcal {D}} \triangleq \frac{\left\| {\mathcal {P}}_{\Omega ^c} \left( {\mathbf {M}} - \widehat{{\mathbf {M}}} \right) \right\| _F ^2}{N \, T \, \overline{\left| \Omega ^c \right| }}. \end{aligned}$$

**Fig. 3:**
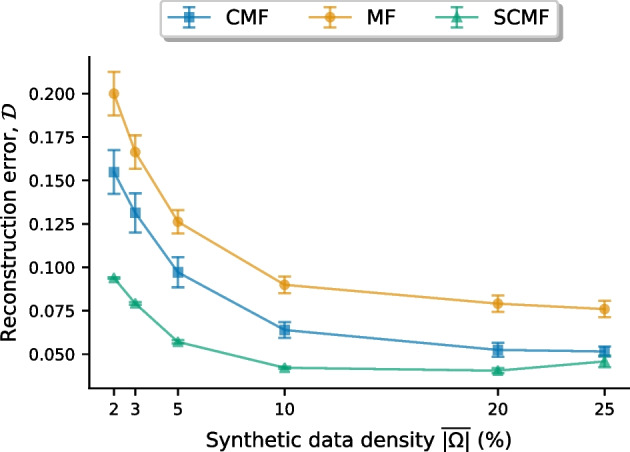
Reconstruction error on synthetic data. Comparing the reconstruction error (${\mathcal {D}}$ by (1)) from different factorization models specified in Table 3 over varying data density ($\overline{\left| \Omega \right| }$). The factorization models are Matrix Factorization (MF), Convolutional MF (CMF) and Shifted CMF (SCMF)

The operator ${\mathcal {P}}_{\Omega ^c} :{\mathbb {R}}^{N \times T} \rightarrow {\mathbb {R}}^{N\times T}$ projects onto unobserved entries and $\overline{\left| \Omega ^c \right| }$ is the fraction of entries from ${\mathbf {Y}}$ in $\Omega ^c$. Figure 3 shows the reconstruction error for factorization models MF, CMF and SCMF over varying data density $\overline{\left| \Omega \right| }$ given as the fraction of observed entries.

Figure 3 indicates that the temporal regularisation used in CMF produces more accurate data reconstructions than the regularisation used in MF as reconstruction error is consistently smaller for CMF than for MF. Moreover, the shift mechanism in SCMF, exploiting correlations between screening histories, gives even smaller reconstruction errors.

**Fig. 4:**
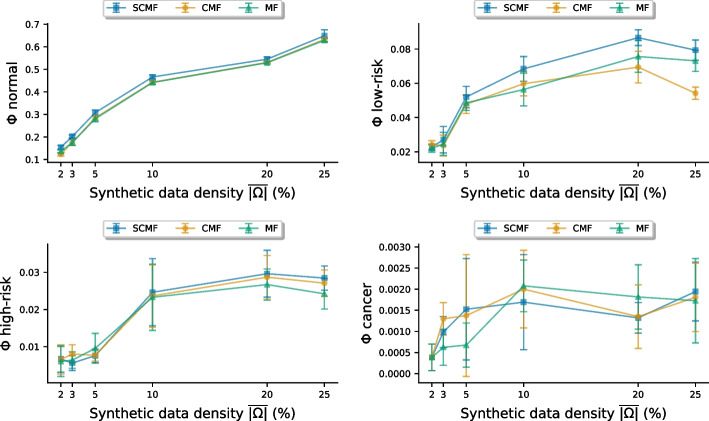
Probability of agreement on synthetic data. Classification performance on synthetic data of varying data density $\overline{\left| \Omega \right| }$. Model performance is given as the *probability of agreement* [13] score ($\Phi _s$ from (8)) with $95 \%$ CI. Higher $\Phi _s \in [0, 1]$ signifies better model fit. The prediction models are Matrix Factorization (MF), Convolutional MF (CMF) and Shifted CMF (SCMF)

In Fig. 4 we compare performance scores, $\Phi _s$ (Eq. (8)) for different models, indicating the *probability of agreement* [13] between hold-out data and predictions. Predicting based on Eq. (5), we required at least two results to be observed prior to the prediction time and in addition we used a moving window to ensure that no result was observed within two years from the time to predict.

The PoA-based scores in Fig. 4 shows that SCMF typically achieves the strongest performance, followed by CMF, mostly outperforming MF. Especially in classifying normal and low-risk, where the number of cases is higher than for high-risk and cancer, the SCMF and CMF attain the highest scores.

### Screening data experiments

We randomly sampled two sets of 15K screening histories (training and test) with at least 3 results between 1991–2015 from the NCCSP data including over 1.7 million female participants. Each selected female was born between 1965–1970 and had her first screening at age 25 (the recommended age to start screening by NCCSP guidelines) to minimize left-censoring. Organizing the histories as sparse time series and combining them produced training and test matrices, each with about $8 \%$ observed entries.

The training histories were used to estimate latent state profiles with the models from Table 3 and a GDL based on [10]. Classification thresholds were obtained by solving (6). The test histories were used for model performance evaluation by comparing observed and predicted results over time, like in experiments on synthetic data. Table 1 gives the normalized PoA score ($\Phi _s$; Eq. (8)) per prediction model.

**Table 1:** Classification performance on registry screening data. Model performance is given as the *probability of agreement* [13] score ($\Phi _s$) with $95 \%$ CI

	$$\Phi _s$$				
Model	Normal	Low-risk	High-risk	Cancer	$\sum \Phi _s$
GDL	$0.35 \left[ 0.32, 0.43 \right]$	$0.087 \left[ 0.077, 0.094 \right]$	$0.15 \left[ 0.13, 0.17 \right]$	$0.47 \left[ 0.44, 0.51 \right]$	1.1
MF	$0.28 \left[ 0.22, 0.35 \right]$	$0.022 \left[ 0.00, 0.063 \right]$	$0.21 \left[ 0.19, 0.24 \right]$	$0.46 \left[ 0.33, 0.54 \right]$	0.98
CMF	$0.31 \left[ 0.23, 0.39 \right]$	$0.11 \left[ 0.063, 0.12 \right]$	$0.29 \left[ 0.27, 0.32 \right]$	$0.77 \left[ 0.72, 0.83 \right]$	1.5
WCMF	$0.31 \left[ 0.26, 0.35 \right]$	$0.25 \left[ 0.23, 0.27 \right]$	$0.27 \left[ 0.24, 0.31 \right]$	$0.78 \left[ 0.73, 0.87 \right]$	1.6
SCMF	$0.33 \left[ 0.27, 0.39 \right]$	$\mathbf {0.59 \left[ 0.57, 0.62 \right] }$	$\mathbf {0.35 \left[ 0.32, 0.37 \right] }$	$0.63 \left[ 0.55, 0.71 \right]$	1.9
SWCMF	$\mathbf {0.36 \left[ 0.29, 0.41 \right] }$	$0.50 \left[ 0.47, 0.51 \right]$	$0.33 \left[ 0.24, 0.41 \right]$	$\mathbf {0.86 \left[ 0.80, 0.90 \right] }$	$\mathbf {2.1}$

The strongest performance per state is indicated in bold

Higher $\Phi _s \in [0, 1]$ signifies better model fit

The overall PoA score in Table 1 was highest for SWCMF from being the most accurate model to predict normal ($\Phi _s = 0.36$) and cancer ($\Phi _s = 0.86$). High-risk and low-risk was best predicted by SCMF ($\Phi _s = 0.35$ and $\Phi _s = 0.59$). Note that CMF improves on MF and both shifted models (SWCMF and SCMF) mostly outperformed their non-shifted variants.

Based on achieving the highest overall PoA score, we study SWCMF in classifying with a forecast horizon ranging from 0.5–5 years. The SWCMF performances from predicting with all data within a given time from the target being removed are given in Table 2.

**Table 2:** Classification performance for Shifted Weighted Convolutional Matrix Factorization over varying forecast horizon as the *probability of agreement* [13] score ($\Phi _s$ from 8) with $95 \%$ CI

	$$\Phi _s$$				
Forecast (years)	Normal	Low-risk	High-risk	Cancer	$\sum \Phi _s$
0.5	$0.35 \left[ 0.26, 0.40 \right]$	$0.61 \left[ 0.52, 0.63 \right]$	$0.21 \left[ 0.18, 0.24 \right]$	$0.91 \left[ 0.86, 0.95 \right]$	2.1
1	$0.32 \left[ 0.25, 0.36 \right]$	$0.59 \left[ 0.56, 0.62 \right]$	$0.45 \left[ 0.35, 0.52 \right]$	$0.90 \left[ 0.83, 0.96 \right]$	2.3
2	$0.36 \left[ 0.29, 0.41 \right]$	$0.50 \left[ 0.47, 0.51 \right]$	$0.33 \left[ 0.24, 0.41 \right]$	$0.86 \left[ 0.80, 0.90 \right]$	2.1
3	$0.38 \left[ 0.33, 0.43 \right]$	$0.40 \left[ 0.38, 0.41 \right]$	$0.24 \left[ 0.21, 0.26 \right]$	$0.79 \left[ 0.70, 0.85 \right]$	1.8
5	$0.20 \left[ 0.086, 0.29 \right]$	$0.024 \left[ 0.020, 0.025 \right]$	$0.20 \left[ 0.10, 0.28 \right]$	$0.68 \left[ 0.66, 0.73 \right]$	1.1

Higher $\Phi _s \in [0, 1]$ signifies better model fit

Table 2 shows that the SWCMF performance is relatively stable up to 3 year forecasts, which is the longest recommended exam interval. However, the performance drops noticeably at the 5 year forecast.

**Fig. 5:**
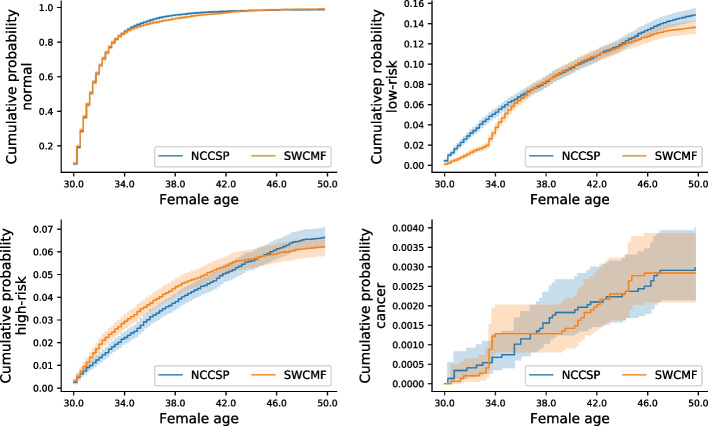
Kaplan-Meier estimates. Comparing Kaplan-Meier estimates from 2 year predictions with Shifted Weighted Convolutional Matrix Factorization (SWCMF) and a hold-out set of registry data from the Norwegian Cervical Cancer Screening Programme (NCCSP)

Plotting the Kaplan-Meier estimates for the hold-out set and the 2 year SWCMF predictions in Fig. 5 indicates a good overall fit as model predictions clearly correlate with the observed data. Note that the *y*-axis scale differs between the plots.

In Fig. 5, the normal rate is slightly underestimated over ages 34–42, as well as the low-risk rate for younger (ages 30–36) and older (ages 44–50) females. These 3 regions correspond well to the times when high-risk is overestimated, which is likely the result of our method for setting the probability thresholds by solving (6). Using time-varying probability thresholds could potentially improve the results here.

**Fig. 6:**
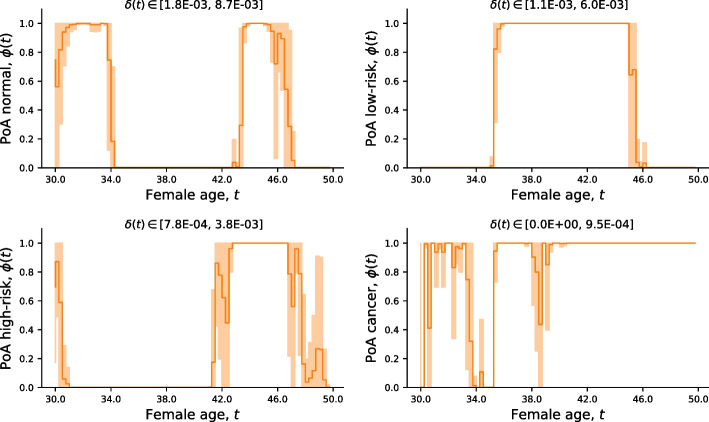
Probability of agreement. The probability of agreement ((PoA); $\phi (t)$ from (7)) between the Kaplan-Meier estimates in Fig. 5. Higher $\phi (t) \in [0, 1]$ means stronger agreement. The range of equivalence margins is given as $\delta (t) \in [a, b]$

The PoA curves from Kaplan-Meier estimates in Fig. 5 are plotted in Fig. 6 to evaluate their agreement.

According to Fig. 6 there is a strong agreement between the cancer estimates, especially after around age 40. As observed in Fig. 5, the drop in PoA for high-risk is complementary to the PoA for normal and low-risk, in which case overestimating high-risk leads to underestimating low-risk and normal in our classification model.

## Discussion and conclusions

Deriving risk prediction models from existing cancer screening registries is a step towards more personalized screening. Here we present a matrix factorization framework that, to our knowledge, is the first approach to use this method for classifying females by the time-varying risk of being diagnosed with cervical cancer from only their current screening histories.

Here we used screening histories from females participating in the Norwegian Cervical Cancer Screening Programme (NCCSP) between 1991–2015, and represent these as sparse time-series vectors fitted into a single matrix. Comparing different algorithms for estimating complete screening profiles for each female we found that the proposed framework, accounting for temporal dependencies within histories and correlations between samples, gave the most accurate estimates.

To illustrate the potential for developing risk prediction models for more personalized screening recommendations, we validated the framework on the NCCSP registry data using Kaplan-Meier (K-M) estimates from model predictions and a hold-out set. The K-M curves showed a strong correlation and a corresponding high *probability of agreement* (PoA) [13] using an equivalence margin $(-\delta (t), \delta (t))$ based the time-varying standard deviation of the ground truth K-M curve.

A typical choice to check if two quantities are within $q \, \%$ of each other is $\delta = q / 100$, but this fixed margin does not permit potential temporal variation in the similarity measure depending on the uncertainty in the reference data. Using the time-varying standard deviation for margin, as in our case, gives a more strict measure if the uncertainty in the ground truth K-M estimate is small but may potentially increase the PoA if this estimate has high variance As the choice for $\delta$ greatly affects the PoA measure, methods for selecting this parameter in cervical cancer screening contexts should be addressed in future work.

Adapting screening recommendations to females at reduced or elevated risk may improve efficiency and precision of cancer screening programs. Prediction models for the individual risk can assist screening programs in adapting to such personalized strategies. The framework presented herein demonstrates the potential for using matrix factorization to derive prediction models for personalized risk estimation based on individual screening data. We also believe that our approach could be applied to data from other types of mass-screening programmes such as breast, colorectal and prostate cancer, which we plan to investigate in future work.

## Methods

We represent the cervical cancer screening data as a partially observed matrix ${\mathbf {Y}} \in {\mathbb {N}}^{N \times T}$. Each row in ${\mathbf {Y}}$ is a one-dimensional time series for a single screening history and each column represents a 3 months time interval. Based on recommendations of 3 years screening intervals for healthy females, and 3 to 6 months for females at elevated risk, choosing 3 months for the time discretisation of the data provides thus a reasonable compromise between temporal resolution and sparsity of the data. In the following, we denote the set of indices where observations in ${\mathbf {Y}}$ are available by $\Omega \subset \left\{ n \right\} _{n=1}^N \times \left\{ t \right\} _{t=1}^{T}$. Moreover, each observed entry $Y_{n,t} \in {\mathbf {Y}}$, representing a *normal*, *low-risk*, *high-risk* or a *cancer* state, is numerically encoded with integer values $s \in \{1,2,3,4\}$ where 1 is normal and 4 is cancer, as in [7].

### A latent state model for cervical cancer screening data

Our basic assumption is that the discrete observed states $Y_{n,t}$ are possibly inaccurate measurements of a continuous *latent state* $M_{n,t}$ that evolves slowly over time for each female. We take each state $Y_{n,t}$ to be observed with probability based on a Gaussian distribution of mean $M_{n,t}$ and variance $1/2\theta$. The parameter $\theta > 0$ models the reliability of the estimate. Thus,

2 $$\begin{aligned} p(Y_{n,t}=s\mid M_{n,t}) \triangleq C_{M_{n,t}}\exp ( - \theta ( s - M_{n, t} )^2) \end{aligned}$$

for some normalization constant $C_{M_{n,t}}$. With this model we have the maximum likelihood estimate

$$\begin{aligned} \theta ^\star = \frac{\left| \Omega \right| }{2\sum _{(n, t) \in \Omega } \left( Y_{n, t} - M_{n, t} \right) ^2}, \end{aligned}$$

where $\left| \Omega \right|$ is the number of observations in $\Omega$.

**Fig. 7:**
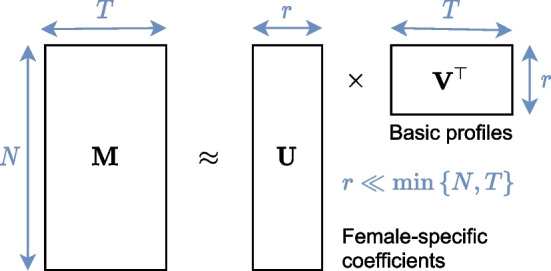
Latent state model for cervical cancer screening data. The matrix ${\mathbf {M}} \in {\mathbb {R}}^{N \times T}$ of latent state profiles decomposed into female-specific coefficients ${\mathbf {U}} \in {\mathbb {R}}^{N \times r}$ and a small number ($r \ll \min \left\{ N, T \right\}$) of basic profiles ${\mathbf {V}} \in {\mathbb {R}}^{T \times r}$

Furthermore, we assume that each latent state profile is a linear combination of a small number of basic profiles ${\mathbf {v}}_1, \dots , {\mathbf {v}}_r$ with $r \ll \min \{N,T\}$. Then the matrix ${{\textbf {M}}}$ of all such profiles can be approximately decomposed as ${{\textbf {M}}} \approx {\mathbf {U}}{\mathbf {V}}^\top$ with ${\mathbf {V}} \in {\mathbb {R}}^{T \times r}$ being the collection of basic profiles and ${\mathbf {U}} \in {\mathbb {R}}^{N\times r}$ being the female-specific coefficients. Figure 7 illustrates the latent state model.

For the simultaneous reconstruction of ${\mathbf {U}}$ and ${\mathbf {V}}$, we propose the variational method of solving

3 $$\begin{aligned} \min _{\begin{array}{c} {\mathbf {U}}, {\mathbf {V}} \end{array}} \left\{ \left\| {\mathbf {W}} \odot \left( {\mathbf {Y}} - {\mathbf {U}} {\mathbf {V}}^\top \right) \right\| _F^2 + \alpha _1 \left\| {\mathbf {U}} \right\| _F^2 + \alpha _2 \left\| {\mathbf {V}} \right\| _F^2 + \alpha _3 \left\| {\mathbf {R}} {\mathbf {V}} \right\| _F^2 \right\} . \end{aligned}$$

Here, ${\mathbf {W}} \in {\mathbb {R}}^{N\times T}$ sets all matrix entries $({\tilde{n}}, {\tilde{t}}) \notin \Omega$ to 0 and multiplies the error over the predicted values at the observed entries $(n, t) \in \Omega$ with some weights $W_{n, t} > 0$. These weights provide a flexible way to incorporate additional information such as uncertainties in exam results and adjusting for entries $Y_{n, t}$ not missing at random with inverse propensity weighting [14]. The matrix ${\mathbf {R}} \in {\mathbb {R}}^{N\times N}$ is used to enforce some time-regularity on the basic profiles ${\mathbf {v}}_1, \cdots , {\mathbf {v}}_r$. We consider two choices of ${\mathbf {R}}$, the first being the forward difference matrix ${\mathbf {R}} = {\mathbf {D}}$. This has the effect of enforcing a high temporal smoothness and is in line with the approach of [12]. As an alternative, we propose ${\mathbf {R}} = {\mathbf {K}}{\mathbf {D}}$ with the forward difference matrix ${\mathbf {D}}$ and ${\mathbf {K}}$ being the Toeplitz matrix with entries $K_{ij} = \exp (- \gamma \, |i-j|)$. This leads to a weaker penalisation of the profiles at faster scales and consequently allows for a larger local variability. The same variability is also observed in the NCCSP data as long intervals with normal results followed by rapid recurrent exams after an abnormal result is detected.

In the NCCSP data we also observe strong correlations between screening histories although as slightly shifted in time. To better exploit these correlations, we extend (3) with female-specific shift matrices ${\mathbf {Z}}_n \in \left\{ 0, 1 \right\} ^{T \times T}$ containing ones in the $z_n$-th diagonal and zeros everywhere else. Now ${\mathbf {V}}^\top {\mathbf {Z}}_n$ shifts the basic profiles $z_n \in {\mathbb {Z}}$ time points either forward ($z_n > 0$) or backward ($z_n < 0$) to improve alignment with screening history ${\mathbf {Y}}_n$. We limit $z_n$ to at most 3 years shift forward or backward in time. To simultaneously optimize ${\mathbf {U}}$, ${\mathbf {V}}$ and the vector ${\mathbf {z}}$ of *N* offset values, we solve

4 $$\begin{aligned} \begin{aligned} \min _{\begin{array}{c} {\mathbf {U}}, {\mathbf {V}}, {\mathbf {z}} \end{array}} \left\{ \sum _{n =1}^N \left\| {\mathbf {W}}_n \odot \left( {\mathbf {Y}}_n - {\mathbf {U}}_n{\mathbf {V}}^\top {\mathbf {Z}}_n \right) \right\| _F^2 \right.&\left. + \beta _1 \sum _{n=1}^N \left\| {\mathbf {U}}_n \right\| _2^2 \right. \\&\left. + \beta _2 \left\| {\mathbf {V}} \right\| _F^2 + \beta _3 \left\| {\mathbf {R}} {\mathbf {V}} \right\| _F^2 \right\} . \end{aligned} \end{aligned}$$

Here ${\mathbf {W}}_n$, ${\mathbf {Y}}_n$ and ${\mathbf {U}}_n$ are vectors from the *n*-th row of each matrix.

Following [12], we optimize (3) by alternating between solving for ${\mathbf {U}}$ at fixed ${\mathbf {V}}$, and solving for ${\mathbf {V}}$ at fixed ${\mathbf {U}}$. To optimize ${\mathbf {z}}$ in (4) we add an exhaustive search over candidate $z_n$. In numerical experiments, we initialize the iterations with $V_{t, k} \sim {\mathcal {N}}(0, 1)$ and ${\mathbf {z}}$ as a vector of zeros. The iterations abort once the relative difference between consecutive estimates $\mathbf {{\widehat{M}}}^{(i + 1)}$ and $\mathbf {{\widehat{M}}}^{(i)}$ is less than $10^{-6}$.

Based on the models (3) and (4), we define five factorization models used in numerical experiments. Table 3 characterizes the factorization models by temporal smoothness model ${\mathbf {R}}$, discrepancy weights $W_{n, t} \in {\mathbf {W}}$ and female-specific shifts $z_n$.

**Table 3:** Matrix factorization models ((3) and (4)) used in numerical experiments

Model name	${R}$	$W_{n, t} : (n, t) \in \Omega$	$\max z_n$ (years)
Matrix Factorization (MF)	${{D}}$	1	–
Convolutional MF (CMF)	${{K}}{{D}}$	1	–
Shifted CMF (SCMF)	${{K}}{{D}}$	1	3
Weighted CMF (WCMF)	${{K}}{{D}}$	${\widehat{p}}(s \mid \epsilon ) \, / \, {\widehat{p}}((n, t) \in \Omega )$	–
Shifted WCMF (SWCMF)	${{K}}{{D}}$	${\widehat{p}}(s \mid \epsilon ) \, / \, {\widehat{p}}((n, t) \in \Omega )$	3

As specified in Table 3, the weights in WCMF and SWCMF incorporate inverse propensity weighting. for our experiments, we derived propensity estimates ${\widehat{p}}((n, t) \in \Omega )$ using the method in [15] and uncertainties in the medical the exam types (i.e., cytology or histology) from [16].

### Predicting the next screening result

To evaluate the proposed framework, we compare here Kaplan-Meier estimates from model predictions with a hold-out set. In future work we plan to evaluate our method for the prediction of individual results.

To predict the future state of a single female, we assume that we are given her current screening record ${\mathbf {x}} \in {\mathbb {N}}^T$ with observations at times $t_0 \le t_p, \ldots , t_q < T$, and that ${\mathbf {m}} \in {\mathbf {M}}$ is the latent state profile underlying ${\mathbf {x}}$. To predict a future state *s* at $t_{q+1} > t_q$, we consider the conditional probability

$$\begin{aligned} p(x_{t_{q+1}} =s \mid {\mathbf {x}}) \propto \int p(x_{t_{q+1}} =s \mid {\mathbf {m}}) \, p({\mathbf {m}} \mid {\mathbf {x}}) \, d{\mathbf {m}}. \end{aligned}$$

Here $p(x_{t_{q+1}} =s \mid {\mathbf {m}})$ corresponds to model (2) and $p({\mathbf {m}} \mid {\mathbf {x}}) \propto p({\mathbf {x}} \mid {\mathbf {m}}) \pi ({\mathbf {m}})$ requires a prior $\pi ({\mathbf {m}})$ for profile ${\mathbf {m}}$. In our approach, we use the empirical distribution of $\mathbf {{\widehat{M}}}$ as a proxy for the true distribution $\pi ({\mathbf {m}})$. This yields the estimated conditional probabilities

5 $$\begin{aligned} \begin{aligned} {\widehat{p}}(x_{t_{q+1}} = s \mid {\mathbf {x}})&\propto \sum _{n=1}^N C_{{\widehat{M}}_{n, t_{q+1}}} \exp (-\theta (s-{\widehat{M}}_{n, t_{q+1}})^2)\\&\times \prod _{j=p}^q C_{{\widehat{M}}_{n,t_j}}\exp (-\theta (x_{t_j}-{\widehat{M}}_{n,t_j})^2). \end{aligned} \end{aligned}$$

Applying estimator 5 to each value $s \in \{1,2,3,4\}$ gives a comprehensive probabilistic overview of a female’s risk. To classify a female into a state from these risk estimates, we consider probability thresholds $\tau = \left\{ \tau _s \in (0, 1) \right\} _{s= 2}^4$ as a way to alleviate the impact of data imbalance. Recall that in the registry data, the states are heavily skewed towards normal, which dominates the risk inference and bias predictions towards the normal state. For each state *s*, we check if the condition ${\widehat{p}}(x_{t_{q+1}} = s \mid {\mathbf {x}}) \ge \tau _{s}$ holds – in which case we predict $x_{t_{q+1}} = s$. The states are evaluated in order from $x_{t_{q+1}} = 4$ down to $x_{t_{q+1}} = 2$. This means that if the condition is satisfied for cancer ($s = 4$), we classify the female into a cancer state and ignore the probabilities of high-risk and low-risk. If neither of the conditions are satisfied we predict normal ($x_{t_{q+1}} = 1$).

To select probability thresholds we first construct Kaplan-Meier estimates ${\widehat{S}}_s$ for each state from model predictions and the corresponding estimates $S_s$ from the ground truths. An event in the Kaplan-Meier estimate is taken to be the first encounter of a specific state in the screening history of a female; if there are several events, we only record the first one. In the second step we solve

6 $$\begin{aligned} \min _{\tau } \sum _{s} \int _{t_0}^T | S_s(t) - {\widehat{S}}_s(t) | \, dt \end{aligned}$$

to obtain the threshold values. Here we use the *differential evolution algorithm* [17] to search for threshold values although an exhaustive search could improve performance at the cost of higher computational complexity. The choice to minimize $| S_s(t) - {\widehat{S}}_s(t) |$ comes from our measure of model performance specified in the next section.

### Model performance evaluation

As a way to assess the potential for developing prediction models for more personalized cervical cancer screening, we validate numerical models over a female cohort. We measure model performance as the *probability of agreement* (PoA) [13] between Kaplan-Meier estimates derived from model predictions and a holdout-set of screening data. This method relies on an appropriate choice of an indifference region $(-\delta , \delta )$ to determine the similarity between the two estimates.

At time $t \in [t_0, T]$ the PoA evaluates to

7 $$\begin{aligned} \phi _s(t) \triangleq p\bigl ( | S_s(t) - {\widehat{S}}_s(t) | \le \delta _s(t)\bigr ). \end{aligned}$$

Here $\phi _s(t)$ is the probability that the distribution of $S_s(t) - {\widehat{S}}(t)_s$ is contained within $\pm \delta$ to support a conclusion about the similarity of the true survival functions. A higher $\phi _s(t)$ implies that $S_s$ and ${\widehat{S}}_s$ are more similar. Currently lacking scientific support for an indifference region eligible in cervical cancer screening, we simply let $\delta (t) = 2 \, {\widehat{\sigma }}(S_s(t))$ estimated from 1000 bootstrap samples.

To quantify model performance in a single number, we estimate the normalized area under the PoA curve

8 $$\begin{aligned} \Phi _s \triangleq \frac{1}{T - t_0} \int _{t_0}^T \phi _s(t) \, dt. \end{aligned}$$

Here $\Phi _s \in [0, 1]$ where $\Phi _s = 1$ indicates perfect model fit. We use the estimate in (8) to compare different models in numerical experiments.

## Data Availability

The cervical cancer screening datasets used in this study can be made available from the Cancer Registry of Norway pursuant the legal requirements mandated by the European GDPR, Article 6 and 9. The data are not publicly available due to individual privacy and ethical restrictions. Source code (Python™) for synthetic data and numerical models can be provided by the corresponding author.

## Abbreviations

CI: Confidence interval
CMF: Convolutional matrix factorization
GDL: Geometric deep learning
K-M: Kaplan-Meier
MC: Matrix completion
MF: Matrix factorization
NCCSP: Norwegian cervical cancer screening program
NN: Nearest neighbour
PoA: Probability of agreement
SCMF: Shifted convolutional matrix factorization
SWCMF: Shifted weighted convolutional matrix factorization
WCMF: Weighted convolutional matrix factorization
